# Supplementary material for: Why maternal continuum of care remains low in Northwest Ethiopia? A multilevel logistic regression analysis
Source: PLoS One. 2022 Sep 19;17(9):e0274729. doi: 10.1371/journal.pone.0274729 (PMC9484641; doi:10.1371/journal.pone.0274729)

PLOS ONE

Why maternal continuum of care remains low in Northwest Ethiopia? a multilevel logistic regression analysis

--Manuscript Draft--

**Manuscript Number:**	PONE-D-21-38409
**Article Type:**	Research Article
**Full Title:**	Why maternal continuum of care remains low in Northwest Ethiopia? a multilevel logistic regression analysis
**Short Title:**	Maternal continuum of care is low in Northwest Ethiopia: a multilevel analysis
**Corresponding Author:**	Tesfahun Hailemariam<br>UOG: University of Gondar<br>ETHIOPIA
**Keywords:**	Antenatal care; skilled birth attendants; postnatal care; continuum of care; Ethiopia
**Abstract:**	Background<br><br>Non-adherence to the maternal continuum of care is still an intractable challenge. Though early initiation and continuum of care are recommended for mothers' and newborns' well-being, empirical evidence indicates that the level of drop-out in the maternal continuum of care is not well known in Ethiopia. This study aimed to assess the level of women's completion of the maternal continuum of care and the factors affecting it in Northwest Ethiopia.<br><br>Methods<br><br>A community-based cross-sectional study was conducted from October 20 to November 28, 2020. Data were collected from 811 women who had a recent history of birth within the last 12 months. The random and fixed effects were reported using an adjusted odds ratio with a 95% confidence interval. The p-value of 0.05 was used to declare significantly associated factors with women's completion of the maternal continuum of care.<br><br>Results<br><br>The study revealed that 6.9% (95%CI: 5.3–8.9%) of women were retained fully on the continuum of maternal care, while 13.2% of women did not receive any care from the existing healthcare system. Membership in the Women's Development Army (AOR = 3.15; 95%CI: 1.25,7.89); being insured (AOR = 2.59; 95%CI: 1.33,5.01); health education (AOR = 2.44; 95%CI: 1.33,4.45); short distance to health facility (AOR = 4.81; 95%CI: 1.55,14.95); and mass-media exposure (AOR = 2.39; 95%CI: 1.11,5.15) were significantly associated predictors of maternal continuum of care.<br><br>Conclusions<br><br>The maternal continuum of care is low in rural northwest Ethiopia. Women education secondary and above, being member of women's development army, being insured, health education, distance, and mass-media exposure were contributing factors to completing the maternal continuum of care. The existing health system should consider the findings of this study to be more effective in improving maternal health programs.
**Order of Authors:**	Tesfahun Hailemariam
	Asmamaw Atnafu
	Lemma Derseh Gezie
	Binyam Tilahun
**Additional Information:**	

Powered by Editorial Manager® and ProduXion Manager® from Aries Systems Corporation

Question	Response
**Financial Disclosure**<br><br>Enter a financial disclosure statement that describes the sources of funding for the work included in this submission. Review the submission guidelines for detailed requirements. View published research articles from *PLOS ONE* for specific examples.<br><br>This statement is required for submission and **will appear in the published article** if the submission is accepted. Please make sure it is accurate.<br><br>**Unfunded studies**<br>Enter: *The author(s) received no specific funding for this work.*<br><br>**Funded studies**<br>Enter a statement with the following details:<br>• Initials of the authors who received each award<br>• Grant numbers awarded to each author<br>• The full name of each funder<br>• URL of each funder website<br>• Did the sponsors or funders play any role in the study design, data collection and analysis, decision to publish, or preparation of the manuscript?<br>• **NO** - Include this sentence at the end of your statement: *The funders had no role in study design, data collection and analysis, decision to publish, or preparation of the manuscript.*<br>• **YES** - Specify the role(s) played.<br><br>* typeset	Doris Duke Charitable Foundation under grant number 2017187.
**Competing Interests**<br><br>Use the instructions below to enter a competing interest statement for this submission. On behalf of all authors, disclose any competing interests that could be perceived to bias this work—acknowledging all financial support and any other relevant financial or non-financial competing interests.	The authors declare that they have no competing interests.

This statement is required for submission and **will appear in the published article** if the submission is accepted. Please make sure it is accurate and that any funding sources listed in your Funding Information later in the submission form are also declared in your Financial Disclosure statement.<br><br>View published research articles from *PLOS ONE* for specific examples.<br><br>**NO authors have competing interests**<br><br>Enter: *The authors have declared that no competing interests exist.*<br><br>**Authors with competing interests**<br><br>Enter competing interest details beginning with this statement:<br><br>*I have read the journal's policy and the authors of this manuscript have the following competing interests: [insert competing interests here]*<br><br>* typeset	
**Ethics Statement**<br><br>Enter an ethics statement for this submission. This statement is required if the study involved:<br><br>• Human participants<br>• Human specimens or tissue<br>• Vertebrate animals or cephalopods<br>• Vertebrate embryos or tissues<br>• Field research<br><br>Write "N/A" if the submission does not require an ethics statement.<br><br>General guidance is provided below. Consult the submission guidelines for detailed instructions. **Make sure that all information entered here is included in the Methods section of the manuscript.**	Not applicable

Powered by Editorial Manager® and ProduXion Manager® from Aries Systems Corporation

**Format for specific study types**<br><br>**Human Subject Research (involving human participants and/or tissue)**<br>• Give the name of the institutional review board or ethics committee that approved the study<br>• Include the approval number and/or a statement indicating approval of this research<br>• Indicate the form of consent obtained (written/oral) or the reason that consent was not obtained (e.g. the data were analyzed anonymously)<br><br>**Animal Research (involving vertebrate animals, embryos or tissues)**<br>• Provide the name of the Institutional Animal Care and Use Committee (IACUC) or other relevant ethics board that reviewed the study protocol, and indicate whether they approved this research or granted a formal waiver of ethical approval<br>• Include an approval number if one was obtained<br>• If the study involved *non-human primates*, add *additional details* about animal welfare and steps taken to ameliorate suffering<br>• If anesthesia, euthanasia, or any kind of animal sacrifice is part of the study, include briefly which substances and/or methods were applied<br><br>**Field Research**<br><br>Include the following details if this study involves the collection of plant, animal, or other materials from a natural setting:<br>• Field permit number<br>• Name of the institution or relevant body that granted permission	
**Data Availability**<br><br>Authors are required to make all data underlying the findings described fully available, without restriction, and from the time of publication. PLOS allows rare exceptions to address legal and ethical concerns. See the PLOS Data Policy and FAQ for detailed information.	Yes - all data are fully available without restriction

Powered by Editorial Manager® and ProduXion Manager® from Aries Systems Corporation

A Data Availability Statement describing where the data can be found is required at submission. Your answers to this question constitute the Data Availability Statement and **will be published in the article**, if accepted.<br><br>**Important:** Stating 'data available on request from the author' is not sufficient. If your data are only available upon request, select 'No' for the first question and explain your exceptional situation in the text box.<br><br>Do the authors confirm that all data underlying the findings described in their manuscript are fully available without restriction?	
**Describe where the data may be found in full sentences. If you are copying our sample text, replace any instances of XXX with the appropriate details.**<br><br>• If the data are **held or will be held in a public repository**, include URLs, accession numbers or DOIs. If this information will only be available after acceptance, indicate this by ticking the box below. For example: *All XXX files are available from the XXX database (accession number(s) XXX, XXX.*).<br>• If the data are all contained **within the manuscript and/or Supporting Information files**, enter the following: *All relevant data are within the manuscript and its Supporting Information files.*<br>• If neither of these applies but you are able to provide **details of access elsewhere**, with or without limitations, please do so. For example:<br><br>*Data cannot be shared publicly because of [XXX]. Data are available from the XXX Institutional Data Access / Ethics Committee (contact via XXX) for researchers who meet the criteria for access to confidential data.*<br><br>*The data underlying the results presented in the study are available*	The data underlying the results presented in the study are available from the corresponding author upon a reasonable request.

Powered by Editorial Manager® and ProduXion Manager® from Aries Systems Corporation

from (include the name of the third party and contact information or URL).<br>• This text is appropriate if the data are owned by a third party and authors do not have permission to share the data.<br><br>* typeset	
Additional data availability information:	

Manuscript Click here to access/download;Manuscript;Manuscript_PO.docx

Why maternal continuum of care remains low in Northwest Ethiopia? a multilevel logistic regression analysis

Tesfahun Hailemariam[1,2]*, Asmamaw Atnafu[3], Lemma Derseh Gezie[4], Binyam Tilahun[1]

Authors' affiliation

[1]Department of Health Informatics, Institute of Public Health, College of Medicine and Health Sciences, University of Gondar, Gondar, Ethiopia.

[2]Department of Health Informatics, College of Health Science, Hawassa, Ethiopia.

[3]Department of Health System and Policy, Institute of Public Health, College of Medicine and Health Sciences, University of Gondar, Gondar, Ethiopia.

[4]Department of Epidemiology and Biostatistics, Institute of Public Health, College of Medicine and Health Sciences, University of Gondar, Gondar, Ethiopia.

Email address:

TH: tesfahunhailemariam@gmail.com, *Corresponding author

AA: asme2002@gmail.com

LD: lemmagezie@gmail.com

BT: binigcms@gmail.com

Abstract

Background: Non-adherence to the maternal continuum of care is still an intractable challenge. Though early initiation and continuum of care are recommended for mothers' and newborns' well-being, empirical evidence indicates that the level of drop-out in the maternal continuum of care is not well known in Ethiopia. This study aimed to assess the level of women's completion of the maternal continuum of care and the factors affecting it in Northwest Ethiopia.

Methods: A community-based cross-sectional study was conducted from October 20 to November 28, 2020. Data were collected from 811 women who had a recent history of birth within the last 12 months. The random and fixed effects were reported using an adjusted odds ratio with a 95% confidence interval. The p-value of 0.05 was used to declare significantly associated factors with women's completion of the maternal continuum of care.

Results: The study revealed that 6.9% (95%CI: 5.3–8.9%) of women were retained fully on the continuum of maternal care, while 13.2% of women did not receive any care from the existing healthcare system. Membership in the Women's Development Army (AOR = 3.15; 95%CI: 1.25,7.89); being insured (AOR = 2.59; 95%CI: 1.33,5.01); health education (AOR = 2.44; 95%CI: 1.33,4.45); short distance to health facility (AOR = 4.81; 95%CI: 1.55,14.95); and mass-media exposure (AOR = 2.39; 95%CI: 1.11,5.15) were significantly associated predictors of maternal continuum of care.

Conclusions: The maternal continuum of care is low in rural northwest Ethiopia. Women education secondary and above, being member of women's development army, being insured, health education, distance, and mass-media exposure were contributing factors to completing the maternal continuum of care. The existing health system should consider the findings of this study to be more effective in improving maternal health programs.

Keywords: Antenatal care, skilled birth attendants, postnatal care, continuum of care, Ethiopia

Background

Every day, about 810 women die due to pregnancy and childbirth-related complications across the world [1]. Annually, 295,000 women die as a result of pregnancy and childbirth-related complications, of which 94% occur in low- and middle-income countries. Statistics show that the lifetime risk of maternal death for a 15-year old is estimated as 1 in every 90 women globally, with 5400 women in developed countries, 37 women in the least developed countries, 45 women in developing countries, and 37 women in Sub-Saharan Africa [1]. Almost all pregnancy-related deaths are associated with complications during pregnancy and childbirth, and nearly 75% of these deaths could be avoided if women were kept on the maternal continuum of care [2]. From global figures, approximately 14,000 maternal deaths and a 1:55 lifetime risk of maternal mortality were reported in Ethiopia [1]. Despite recent progress, Ethiopia continues to suffer from an unacceptable number of maternal deaths [3].

Improving maternal health has been a global public health priority [4, 5]. The maternal continuum of care through pregnancy to postpartum is a rallying call to make a woman's life safe and joyful [6]. It is a proven intervention for maternal mortality reduction and is efficient for making a woman more productive in life [7], as it links a woman to the maternal health care services provision centers such as home, community, and health facilities [6]. The World Health Organization (WHO) suggested at least four antenatal care (ANC) visits and one or more postnatal visits within 2 days of delivery to improve maternal health and birth outcomes [8, 9].

Antenatal care is a vital component of the maternal continuum of care for women and is believed to be the basis for women and newborns to survive and thrive [10, 11]. Early initiation and proper ANC follow-up reduce adverse pregnancy outcomes in both mother and baby since it promotes early identification of pregnancy-related complications and provides an opportunity for a woman to discuss with a health care provider about pregnancy and health

behavior [12]. Health care facility delivery with the assistance of a skilled health care attendant is another strategy for reducing maternal morbidity and mortality [9], as births carried out in homes under unhygienic conditions could lead to a high incidence of maternal death [13]. Receiving postnatal care from a healthcare provider at the recommended time with its appropriate content also prevents complications that could arise after childbirth and helps a mother get comprehensive health education [9].

With regard to the health sector's mission in Ethiopia, a three-tiered healthcare delivery system has been put in place to facilitate the realization of the health system's goals; at the primary level of the healthcare delivery system, health extension workers are designated for consultation and service provision in the areas of family planning, antenatal care, postnatal care, and child health service [14]. Besides, the government's initiatives and commitment towards the maternal continuum of care indicate there are identified intervention areas to strengthen maternal health programs [15]. Despite the certain improvement in maternal healthcare utilization, however, completion of the maternal continuum of care is very low [16] and improving maternal health remains a major challenge for the health system in Ethiopia [3].

According to studies, four out of every ten women did not receive ANC, and seventeen percent of women in Ethiopia had their first prenatal care visit before the fourth month of pregnancy [17] and fifty-six percent of women gave birth in a health facility after receiving four or more antenatal care visits, and seventeen percent received postnatal visits within 48 hours after birth [9].

According to the body of literature, both individual and community-level factors are influencing maternal health care utilization [18-20]. Though there was promising progress in maternal healthcare utilization and it is a prioritized agenda in Ethiopia [21], the available evidence on women's completion of the maternal continuum of care is inconclusive. Understanding the existing point of women's completion of the maternal continuum of care and

identifying contextual factors that limit women's completion of the maternity care continuum helps to be effective in the implementation of maternal health programs. Considering the hierarchical structure of women with less than one year (level 1) nested within a health center (level 2), it is appropriate to predict the point of women's retention in the maternal continuum of care. Therefore, this study aimed to examine individual and community-level factors affecting women's completion of the maternal continuum of care.

Methods

Study design and setting

A community-based cross-sectional study was conducted in 11 clusters in Wogera and Gondar Zuriya districts in the central Gondar zone of Amhara National Regional State from October 20 to November 28, 2020. The districts were located 658km from Addis Ababa, the capital city of Ethiopia. There were 16 health centers and 88 health posts in the districts. The total population was 524, 907 (female=260879 and male=264028) at the time of the survey, of which 122,303 and 16745 were women in the reproductive age group and surviving infants, respectively.

Study population

All women in the study districts who gave birth within the last 12 months prior to data collection were the source population, and randomly selected women who were permanent residents of the selected kebele and willing to participate in the study were the study population.

Sample size determination

For the single population proportion formula, the assumptions used were proportion of focused antenatal care p= 39.9%, proportion of skilled birth attendants after completing antenatal care four and above p=31.1%, and proportion of women retained in the continuum of maternal care p=12.1% [22] and considering 95% CI, margin of error = 5%, design effect of 2 and 10% non-response rate, the sample sizes were 811,726, and 360, respectively. Double population

proportion formulas were considered in estimating different sample sizes, considering factors affecting maternal health service utilization. The sample size was computed using the STAT-CALC program of Epi-info version 7.0. software using the following assumptions: 5% level of significance (two-sided), 80% power, a 1:1 ratio of exposed to non-exposed maternal health service utilization, 49.7% of the outcome in the exposed group and 50.3% of the outcome in the unexposed group, and considering being a model household in the community as a predictor variable to bring change in two populations [23], a design effect of 2, and a 10% non-response rate. Of the different sample sizes estimated, the largest sample size, 811, was obtained from a single population formula and considered in the study.

Sampling procedure

A two-stage sampling technique was employed to reach study participants. Since the districts have fifteen health centers, eleven health centers (clusters) and kebeles at each cluster were selected randomly with systematic sampling. Households were selected from a sampling frame developed using a family folder of health extension workers, which contained a list of all births for rural women. The sampling interval was obtained by dividing the source population by the total sample size estimated. The first woman was chosen from a sampling frame in the family folder using a simple random sampling technique, and the random selection was carried out using the random between functionality in Microsoft Excel. Using this technique, the required sample size was obtained, and a total of 811 eligible women were interviewed for the study and included in the analysis.

Study variables and measurement

Response variable

Women's completion of the maternal continuum of care is categorized as "Yes" if the women received four or more antenatal care visits, skilled birth attendance, and postnatal care visits within 48 hours after birth, and "No" otherwise.

Explanatory variables

Ranges of predictor variables were selected and grouped as individual and community level variables based on previous literature.

Individual level factors

Individual level variables were age of the respondents, marital status of the respondents, occupation, education status of respondents, education status of partner, intendedness of the current pregnancy, distance from home to facility, household wealth index, being a member of community-based health insurance, mass-media exposure, health extension workers' home visits during pregnancy, being a member of Women's Development Army, and getting health education from a nearby health post during pregnancy.

Community level factors

Community and cluster level variables were aggregated from individual and cluster level data such as health facility readiness, the average distance from the village to the health facility, wealth status in the cluster, and mass-media exposure. Health facility readiness for service provision was assessed using the WHO service availability and readiness assessment guideline [24]. Then the aggregated clusters were categorized as low if the proportion of clusters' readiness for service provision was 0–72% whereas high if the proportion was 73–100%. The household wealth index median value was three, and the community value was classified as low if the median value of the community is below three and high otherwise (middle and above). The mass-media exposure status of the community was categorized as low if the proportion of exposure status was 0–17%, and whereas high if the proportion of mass media exposure status in the community was 18–100%, and the walking distance from home to health facility was near if the aggregated proportion was 0–71%, and far if the aggregated proportion of walking distance was 72–100%.

Data collection instrument and procedures

The instrument was developed in English and translated into the local language. Since experts' views were sought for the psychometric properties of the face and content validity, experts were invited to review the relevance of each question in the instrument. The tool was revised according to expertise's view and was piloted out of the selected clusters and validated before data collection. The proportion of the rated item and scale content validity index was computed, and the experts' rated item content validity index and scale level validity index were 0.98 and 0.81, respectively. Data collectors and supervisors were trained on the instrument. The household data were collected using an interviewer-administered questionnaire via face-to-face interviews.

Operational definitions

Maternal continuum of care: It is the continuity of care throughout pregnancy (utilization of antenatal care four and above, skilled birth attendance, and postnatal care within 48 hours after birth) [6].

Data management and statistical analysis

The data were entered into EpiData 4.1 and then exported to STATA 14 for analysis. For wealth index assessment, principal component analysis was employed to reduce data that measure the same construct together, and the data were recoded into binary variables. Variables with low frequencies were combined, and frequencies greater than 95% and less than 5% were excluded from the analysis. Under the component loading, there were twenty-one variables correlated with measuring the wealth index, such as having one's own house, toilet, electricity, kitchen, charcoal, television, radio, modern bed, cotton, mobile phone, farming land, having animals in the house, such as cows, horse, donkey, goat, sheep, chickens, having a separate room for animals, having a beehive, and having a bank account number with deposited Birr (Ethiopian

currency). The wealth quantile was categorized into five categories, such as poorest, poor, middle, richer, and richest.

Bivariable multilevel logistic regression was used for each factor against the outcome variable without controlling the effect of other explanatory variables. For multivariable multilevel logistic regression analysis, factors at both individual and cluster levels with a p-value <0.25 were considered as candidate variables. The fixed-effect of individual and cluster level variables was reported using the Crude Odds Ratio (COR) with a 95% confidence interval.

During model building, four models were built to estimate both the fixed effects of the individual and community-level factors and the random effects between-community variation on the women's completion of the maternal continuum of care. The model I was built without any explanatory predictors to examine the random effect of cluster variation by using Intra-Class Correlation (ICC) to justify the application of multilevel analysis in this study. Therefore, random parameters in this model were used as a benchmark to compare against parameters of successive models (Model II, Model III, and Model IV) by looking at the decline of the ICC value [25]. Model II was built to examine the contributions of individual-level factors. Model III was fitted to determine between-cluster variations. Finally, Model IV (individual and cluster-level factors model) was built by combining both individual and cluster-level factors simultaneously by controlling the effect of other predictors. The measure of association was reported as Adjusted Odds Ratio (AOR) with a 95% CI. The p-value < 0.05 was used to identify factors significantly associated with women's completion of the maternal continuum of care. The random effect was presented using intra-class correlation (ICC); thus, in all models, ICC and its change from the null model were examined. The Proportional Change in Variance (PCV) was computed with reference to the null model to examine the relative contribution of level II, III, and IV predictors in explaining the odds of women completing the maternal continuum of care.

To estimate the goodness-of-fit of the adjusted final model, the Akaike information criteria (AIC) and loglikelihood model were used in comparison with other models. The multicollinearity effect was checked by using the mean of the variation inflation factor (VIF) value at a cut-off point of 10, and it indicated that there was no multicollinearity effect among predictor variables [26]. The interaction effect of the variables was checked by creating a new variable, and the created new variable, or product term, became either statistically significant or not at p-value<0.05.

Ethical considerations

Study approval and ethical clearance were obtained from the University of Gondar ethical review board (R.NO. V/P/RCS/05/2020) and permission was granted from the central Gondar zonal health department. Verbal consent was obtained and participants were informed about the objective, importance of the study, procedure and duration, risk and discomfort, benefits of participating in the study, confidentiality, and the right to refuse or withdraw during data collection. After obtaining the relevant information, participants were counseled on the benefits of attending maternal health care services and the consequences of missing maternal health care services. The COVID-19 protocol was maintained during data collection.

Results

Sociodemographic and reproductive characteristics of the study participants

A total of 811 women participated in the study, with a response rate of 100%. The minimum and maximum ages of the respondents were 15 years and 48 years, with a mean age of 28 years. The majority of participants did not attend education, 507 (61.7%). Most of the participants were housewives in their occupations 801 (98.8%) and married 790 (97.4%). The proportion of women was nearly equal across the wealth quantiles of the household level, with the poorest

163 (20.1%), poorer 162 (20.0%), middle 163 (20.1%), richer 161 (19.8%), and the richest 162 (20.0%) (Table 1).

Table 1: Sociodemographic and reproductive characteristics of study participants in Northwest Ethiopia, 2021(n=811)

Variables	Category	Maternal continuum of care		Total (%)	(p-value)
		Yes (%)	No (%)		
Age group of respondents (years)	15-24	16 (7.0)	211(93.0)	227 (28.0)	0.935
	25-34	28(7.1)	366 (92.9)	394(48.6)	
	35 and above	12 (6.3)	178 (93.7)	190(23.4)	
Marital status	Married	54(6.8)	736(93.2)	790(97.4)	0.632
	Single/divorced/widowed /separated	2(9.5)	19(90.5)	21(2.6)	
Occupation	Housewife	55(6.9)	746(93.1)	801(98.8)	0.698
	Employee/laborer/merchant	1(10.0)	9(90.0)	10(1.2)	
Education level of respondents	Did not attend education	23(4.6)	477(95.4)	507(61.7)	<0.001
	Primary education	17(7.9)	198(92.1)	215(26.5)	
	Secondary and above	16(16.7)	80(83.3)	91(11.8)	
Education level of husbands	Did not attend education	27(5.2)	496 (94.8)	523(64.5)	<0.001
	Primary education	15(7.3)	190(92.7)	205(25.3)	
	Secondary and above	14(16.9)	69(83.1)	83(10.2)	
Intendedness of pregnancy	Yes	49(7.6)	593(92.4)	642(79.2)	0.111
	No	7(4.1)	162(95.9)	169(20.8)	
Distance home to health facility	>=5km	33(5.7)	543(94.3)	576(71.0)	0.039
	<5km	23(9.8)	212(90.2)	235(29.0)	
Household wealth index	Poorest	11(6.7)	152(93.3)	163(20.1)	0.446
	Poorer	6(3.7)	156(96.3)	162(20.0)	
	Middle	13(8.0)	150(92.0)	163(20.1)	
	Richer	12(7.5)	149(92.5)	161(19.8)	

	Richest	14(8.6)	148(91.4)	162(20.0)	
**Being insured**	Yes	41(10.3)	353(89.6)	394(48.6)	<.000
	No	15(3.6)	402(96.4)	417(51.4)	
**Membership in women development army**	Yes	35(13.5)	225(86.5)	260(32.1)	<.000
	No	21(3.8)	530(96.2)	551(67.9)	
**Health extension workers home visiting**	Yes	31(8.6)	330(91.4)	361(44.5)	0.091
	No	25(5.6)	425(94.4)	450(55.5)	
**Mass media exposure**	Yes	15(10.7)	125(89.3)	140(17.3)	0.051
	No	41(6.1)	630(93.9)	671(82.7)	
**Getting health education at health post level**	Yes	29(9.2)	285(90.8)	314(38.7)	0.037
	No	27(5.4)	470(94.6)	497(61.3)	

The proportion of women who intended the current pregnancy was 642 (79.2%). Study participants visited by health extension workers during the current pregnancy were 361 (44.5%) and those who got health education from a nearby health post were 314 (38.7%). Being a member of community-based health insurance and being a member of the women's development army in the kebele was 394 (48.6%) and 260 (32.1%), respectively (Table 1).

Community level characteristics of the study participants

In this study, the wealth index at the community level indicated that 611 (75.3%) of the participants had high wealth status. The proportion of study participants who had high media exposure at the community level was 372 (45.9%). The level of health facility readiness for antenatal care service provision was 619 (76.3%). Among the participants, the larger proportion of respondents 615(75.8%) had a walking distance of less than five kilometers from their home to a nearby health facility. Women's retention in the maternal continuum of care was significantly associated with clusters (health centers) (chi-square = 28.19, p-value = 0.001).

Bivariable multilevel logistic regression analysis indicated that media exposure in the community and distance from home to health facility were significantly associated factors with the maternal continuum of care (Table 2).

Table2: Community level characteristics of respondents in Northwest Ethiopia, 2021 (n=811)

Variables	Category	Maternal continuum of care		Total (%)	(p-value)
		Yes (%)	No (%)		
Health facilities	Ambageorgis	16(15.2)	89(84.8)	105(12.9)	0.001
	Gedebiye	11(11.6)	84(88.4)	95(11.7)	
	Birra	3(5.0)	57(95.0)	60(7.4)	
	Tirgosgie	6(9.0)	61(91.0)	67(8.3)	
	Woybey	5(10.9)	41(89.1)	46(5.7)	
	Dergaj	3(7.1)	39(92.9)	42(5.2)	
	Miniziro	0(0.0)	104(100.0)	104(12.8)	
	Maksegnit	2(2.4)	83(97.6)	85(10.5)	
	Lamba	3(3.8)	77(96.3)	80(9.9)	
	Enfranz	4(4.3)	88(95.7)	92(11.3)	
	Abawarka	3(8.6)	32(91.4)	35(4.3))	
Wealth Index	High	45(8.2)	506(91.8)	551(67.9)	0.039
	Low	11(4.2)	249(95.8)	260(32.1)	
Health facility readiness	High	48(7.8)	571(92.2)	619(76.3)	0.087
	Low	8(4.2)	184(95.8)	192(23.7)	
Mass-media exposure	High	34(9.1)	338(90.9)	372(45.9)	0.021
	Low	22(5.0)	417(95.0)	439(54.1)	
Walking distance from home to health facility	>=5km	4(2.0)	192(98.0)	196(24.2)	0.002
	<5km	52(8.5)	563(91.5)	615(75.8)	

Dropouts of women completion of maternal continuum of care

In this study, women who attended antenatal care four and above, skilled birth attendants, and postnatal care visits within 48 hours after birth were considered during the analysis. The rates

of antenatal care follow-up during pregnancy, antenatal care four and above, and skilled birth attendant after ANC4+ completion were 86.8% (95% CI: 84.3-89.1), 39.6% (95% CI: 36.2-43), and 29.1% (95% CI: 26-32.4), respectively. The study indicated that 13.3% (95% CI: 11.1-15.9) of participants had attended postnatal care within 48 hours after birth. The proportion of women who were retained fully on the maternal continuum of care during pregnancy was 6.9% (95%CI: 5.3-8.9) (Fig 1).

Fig 1. Maternal health service utilization in completion of maternal continuum of care from antenatal care to postnatal care within 48hrs after birth in Northwest Ethiopia, 202).

Multivariable multilevel logistic regression analysis of maternal continuum of care

During analysis, four mixed-effect regression models were built to predict the maternal continuum of care based on the different exposure variables at individual and community levels (Table 3). The intercept-only model was run without any predictors to test the random effect between cluster variation on the maternal continuum of care. An estimate of intraclass correlation was 12% (95% CI: 3%, 35%), implying that 12% of the variation in the maternal continuum of care was due to cluster-level factors and 43.5% was due to differences across clusters, and this variation was significant (= 0.43, p 0.000).

At the individual level (Model II), predictors such as women's education secondary and above, being a member of the women's development army, being a member of community-based health insurance, getting health education at the health post level, distance from home to health facility, and husband education secondary and above were statistically associated with women's completion of the maternal continuum of care. Intraclass correlation in model II indicated that 10% of the difference in women's completion of the maternal continuum of care was attributed to cluster variability.

The finding of this study in model III showed that exposure to mass media and walking distances of less than five kilometers from home to a health facility were statistically significant in women's completion of the maternal continuum of care.

Finally, after controlling all factors, the full model (model IV) was developed, including individual and cluster-level factors simultaneously. The finding indicated that there was a substantial reduction in variance in predicting women's completion of the maternal continuum of care. About 93% of women's completion of the maternal continuum of care in the clusters was explained in the last model where the lowest Akaike information criteria and loglikelihood were observed (362.62 and 168.31, respectively). The odds of adhering to the maternal continuum of care were 3.15 times (AOR = 3.15; 95%CI: 1.25, 7.89) higher among women with secondary and above education as compared to their counterparts. Women who were members of a women's development army in the kebele were 2.91 times (AOR = 2.91; 95%CI: 1.56, 5.44) more likely to complete the maternal continuum of care as compared to women who were not members of a women's development army. Women insured in community-based health schemes were 2.59 times more likely to complete the maternal continuum of care than their counterparts (AOR = 2.59; 95%CI: 1.33, 5.01).

Our study found that women who got health education from a health post during the pregnancy period were 2.44 times (AOR = 2.44; 95%CI: 1.33, 4.45) more likely to complete the maternal continuum of care as compared to women who did not get health education from a nearby health post. Furthermore, women who walked less than five kilometers from home to health facility were 4.81 times (AOR = 4.81; 95%CI: 1.55,14.95) more likely to complete the maternal continuum of care than those who walked more than five kilometers. Finally, women who had been exposed to mass media were 2.39 times (AOR = 2.39; 95%CI: 1.11, 5.15) more likely to complete the maternal continuum of care as compared to their counterparts (Table 3).

Table 3: Multivariable multilevel logistic regression analysis of predictors of women's completion of maternal continuum of care in Northwest Ethiopia, 2021 (n=811)

Variables	Category	COR (95%CI)	Model I (Null model)	Model II AOR (95%CI)	Model III AOR (95%CI)	Model IV AOR (95%CI)
**Women education level**	Did not attend education	1		1		1
	Complete primary education	1.70(0.88,3.28)		1.45 (0.69,3.02)		1.54 (0.74,3.21)
	Complete secondary education and above	3.61(1.78,7.32)		2.93(1.18,7.28)		3.15(1.25,7.89)
**Husband education level**	Did not attend education	1		1		1
	Complete primary education	1.43(0.74,2.78)		0.93(0.43,1.96)		0.87(0.41,1.83)
	Complete secondary education and above	3.48(1.68,7.21)		1.63(0.63,4.18)		1.39(0.53,3.62)
**Being a member in WDA**	Yes	3.84(2.16, 6.83)		2.85(1.53,5.33)		2.91(1.56,5.44)
	No	1		1		1
**HEWs home visiting**	Yes	1.85(1.04,3.28)		1.91(1.03,3.57)		1.69(0.88,3.24)
	No	1		1		
**Being insured**	Yes	3.07(1.65,5.71)		2.52(1.29,488)		2.59(1.33,5.01)
	No	1		1		
	Yes	1.87(1.06,3.29)		2.40(1.314.42)		2.44(1.33,4.45)

**Getting health education**	No	1		1		
**Facility readiness**	Yes	1.66(0.52,5.23)			0.78(0.27, 2.26)	0.54(0.19,1.51)
	No	1			1	1
**Distance from home to health facility**	<5km	4.37(1.23,15.29)			5.09(1.52, 16.99)	4.81(1.55,14.95)
	>=5km	1			1	1
**Mass-media exposure**	High	1.89(0.72,4.98)			2.20(0.96, 5.03)	2.39(1.11,5.15)
	Low	1			1	1
**Random effects**	Variance		0.43	0.37	0.10	0.03
	ICC (%)		12	10	3	0.9
	PCV (%)		ref	14	76	93
	AIC		401.43	365.38	398.52	362.62
	-2Loglikelihood		397.44	345.38	388.52	336.62

Discussion

We conducted a study on women's completion of the maternal continuum of care among women in rural areas of Northwest Ethiopia. One out of every fourteen women completes a maternal continuum of care, while one out of eight women does not receive maternal health care from the existing health system. The final model of multivariable multilevel logistic regression analysis indicates that at the individual level, completing secondary and above education, being a member of the women's development army, getting health education from a health post, and being a member of community-based health insurance, at the community level, distance from home to health facility, and mass-media exposure were significant predictors of women's completion of the maternal continuum of care.

According to our review of literature, the proportion of women completing the maternal continuum of care ranges from 6.4% [27] to 67.8% [28]. The current study was congruent with research studies conducted in Ghana [29, 30], Khammouane, Lao PDR [31], and Pakistan [27]. However, our finding was lower as compared to studies conducted in other parts of Ethiopia [22, 28, 32-36], and elsewhere in Tanzania [37], nine South Asian and sub-Saharan African countries [38], Pakistan [39], Nepal [40], Cambodia [41, 42], the district of Xaybouathong in Lao PDR [43], and Egypt [44].

The deflated proportion of women in the current study as compared to other studies could be attributed to the fact that our study considered women who had received antenatal care visits four and above, skilled birth attendance at birth, and postnatal care visits within 48 hours after birth, whereas others included the post-partum period within six weeks after birth [29, 30, 32, 34, 36, 44]. The larger drop out in women's completion of the maternal continuum of care risks women to unwanted death, as women could miss continuity in maternal healthcare utilization. Another justification may be the low rate of early initiation and completion of antenatal care visits [45-47]. It is believed that early booking of antenatal care creates an opportunity for a woman to get pregnancy-related information such as birth preparedness, danger signs during pregnancy and the postpartum period, nutritional counseling, and complete content of focused antenatal care components and helps with early checkups for medical and obstetric conditions [34, 47-49]. Another possible justification could be that in the current study, the rate of late initiation of ANC (after 16 weeks of pregnancy) was nearly 50%, i.e., one in every two women did not receive an early antenatal care visit, as women have not been provided the aforementioned healthcare benefits of early initiation of antenatal care visits. Therefore, we suggest early booking of ANC and staying on the path of the maternal continuum of care to improve women’s and newborns’ well-being. The low point of women's completion of the maternal continuum of care might be that, though "home delivery free" is the program that has

been launched by the government of Ethiopia, it is still one of the unresolved challenges of maternal health program indicators. For example, the Ethiopian demographic survey of 2016 [9] showed that 73% of births occur at home. In our study, among women who did not receive facility delivery and postnatal care visits, nearly two-thirds of women preferred traditional birth attendants to give birth and did not have awareness about the importance of postnatal care after birth. They perceived that they and their babies were safe and did not intend to go to a health facility. Another reason might be that health-seeking behavior, beliefs, and norms about the place of birth could contribute to a low rate of women's completion of the maternal continuum of care. Literature states that the most common reasons for home delivery are family and relatives influence, usual practice, unexpected labor, and not being sick, depending on traditional healers and spiritual healing, such as prayer [50]. Women's perception that pregnancy is not an illness and that attending ANC is useless could act as a barrier to utilizing health care services [51]. Moreover, unlike other studies [28, 32-34, 36, 38, 41, 43, 44] that reported higher proportions of the maternal continuum of care by using ordinary logistic regression, our study considered multilevel multivariable logistic regression models.

Regarding predictors, our finding revealed that the odds of women's education completing secondary and above had a significant association with women's completion of the maternal continuum of care, which was 3.34 times as compared to their counterparts. This finding is in consonance with the findings of studies conducted in Ethiopia [22, 32, 33, 52] and elsewhere [30, 38, 40, 42, 44]. The possible justification may be that educated women could easily grasp pregnancy-related counseling during health care provision and information through mass media. Moreover, educated women may have a role in self-determination role so that they can decide by themselves to seek healthcare from healthcare providers. In addition, educated women may not be influenced by beliefs and norms. A study conducted in rural Mali on the role of belief and norms for maternal health care utilization revealed that women were expected

to obey their husbands during childbirth in order to have an easy delivery [53]. If the husband orders the woman to stay at home during labor, the woman should obey him and stay at home for home delivery.

In this study, women who were members of the women's development army were 2.91 times more likely to complete the maternal continuum of care as compared to their counterparts. This finding was supported by studies done in Ethiopia [54, 55]. The similarity of our finding might be that an active woman in the development army could get different benefits in the kebele, such as health education about birth preparedness and complication readiness, being linked to health facilities by HEWs at the time of referral, reducing delays that could be related to a pregnant woman, and enhancing harmonized relationships with health extension workers, community, and healthcare facilities.

The odds of women completing the maternal continuum of care were 2.59 times higher among respondents who were members of community-based health insurance as compared to those who were not insured. Our finding was in concord with other studies in Ethiopia [56, 57], Ghana [58], Manzi [59], Nigeria [60], and Tanzania [61]. This consistency of our finding might be due to the commencement of a community-based health insurance scheme in Ethiopia which provides maternal healthcare services for free to all women. Due to the low economic conditions of the country, women could be less motivated to use maternal health care services if they were required to pay for them. Another possible explanation might be that community health insurance reform in Ethiopia could have had an influence on the health-seeking behavior of a woman.

The findings of this study showed that getting health education at the health post level made women 2.44 times more likely to complete the maternal continuum of care as compared to women who did not get health education. Our finding was consonance with a prior study [31] and a study done in Pakistan revealed that facility delivery was higher among women who got

health education from community health workers when compared to those who did not get health education during the pregnancy period [62]. In Ethiopia, frontline health extension workers (HEWs) are commonly tasked with providing maternal and child health services at primary health care units [63]. Health extension workers' health message advocacy for maternity service utilization at the health post level could have an influence on the maternal health care continuum. This finding was reaffirmed by [64], as community health workers have a vital role in facilitating women's completion of maternal continuum care.

Regarding community level distance, the odds of completing the maternal continuum of care were 4.18 times higher in women who had a walking distance of less than five kilometers from home to health facility as compared to women who had a walking distance of greater than five kilometers to reach the health facility. Studies conducted in other parts of Ethiopia supported this finding that less travel time to health facilities predicted completion of the maternal continuum of care [32, 33, 65] and elsewhere [29, 31, 66-68]. This could be because women who live in difficult-to-reach areas or unable to access health facilities may face lack of transportation to health facilities, particularly at night [69] since many times labor begins at night [70] since most women take walks by foot or animal, and lack of transportation and long distances are barriers for maternal healthcare utilization [71]. Another reason might be that women at remote locations could have less awareness and miss a chance to get pregnancy-related information as compared to women who live near a country/health facility. Another possible explanation might be the inaccessibility of health facilities, according to Berhan et al., as women pay around 4000 Birr for less than a hundred kilometers of travel in Ethiopia, which is unfair and too much in the country by any standard [72].

This study found that women who were exposed to the mass media were 2.39 times more likely to complete the maternal continuum of care than those women who were not exposed. This finding is in line with previous studies in Ethiopia (30, 44). According to a study

in Pakistan, about 58% of women with weekly exposure to mass media gave birth in health institutions as compared to 36% of women with less frequent mass-media exposure [62]. The possible explanation is that the mass media has the potential to influence positive behavioral changes in maternal health service utilization because women can easily access maternal and child health-related information at home

Limitation and strength of the study

Recall bias may be the limitation of this study, as the participants might not remember the previous event rather than recent events. Nevertheless, we attempted to specify questions related to the service given during prenatal, natal, and postnatal periods by probing. In our study, we did not exclude women who had previous pregnancy complications; this could have introduced social desirability bias in which respondents could answer questions that they thought would lead to their being accepted. However, during the response, an open-ended approach was used to prevent the participant from simply agreeing or disagreeing.

Conclusions

Women's completion of the maternal continuum of care was extremely low in the study area. There was a substantial reduction in community variation in the final model, indicating that there was a reduced representation of unobserved variables that explain the variation. The findings suggest that women's education, being insured, being a member of the women's development army, health education at the health post level, distance from home to health facility, and women's exposure to mass media are contextual factors contributing to women’s completion of the maternal continuum of care and need attention to improve maternal service utilization. Moreover, maternal health programs should be strengthened, particularly focusing on awareness creation of women about the maternity care continuum, increasing healthcare accessibility, membership advocacy in community-based health insurance and women's

development army, and health education promotion by health extension workers may help women to complete the maternal continuum of care.

Acknowledgements

We thank Central Gondar zone health department, Wogera and Gondar Zuriya district health offices, the University of Gondar Comprehensive Specialized Hospital, and health extension workers for their provision of necessary information and support during data collection. Our gratitude also goes to the study participants, data collectors, and supervisors who took part in the study.

Author Contributions

Conceptualization: TH, TH, AA, LDG, BT

Data curation: TH, TH, AA, LDG, BT

Formal analysis: TH, TH, AA, LDG, BT

Investigation: TH, AA, LDG, BT

Methodology: TH, AA, LDG, BT

Project administration: TH, AA, LDG, BT

Resources: TH, AA, LDG, BT

Software: TH

Supervision: TH, AA, LDG, BT

Validation: TH

Writing – original draft: TH

Writing – review & editing: TH, AA, LDG, BT

References

1. WHO U, UNFPA, World Bank Group and the United Nations Population Division. Geneva: World Health Organization; . Trends in maternal mortality 2000 to 2017: estimates 2019. Licence: CC BY-NC-SA 3.0 IGO.

2. Say L, Chou D, Gemmill A, Tunçalp Ö, Moller A-B, Daniels J, et al. Global causes of maternal death: a WHO systematic analysis. The Lancet Global Health. 2014;2(6):e323-e33.
3. Shiferaw S, Spigt M, Tekie M, Abdullah M, Fantahun M, Dinant G-J. The effects of a locally developed mHealth intervention on delivery and postnatal care utilization; a prospective controlled evaluation among health Centres in Ethiopia. PLoS One. 2016;11(7):e0158600.
4. WHO. MDGs: Health and the Millennium Development Goals. 2005.
5. Sachs J, McArthur J. The millennium project: a plan for meeting the millennium development goals. Lancet. 2005;365:347–53.
6. Kerber KJ, de Graft-Johnson JE, Bhutta ZA, Okong P, Starrs A, Lawn JE. Continuum of care for maternal, newborn, and child health: from slogan to service delivery. The Lancet. 2007;370(9595):1358-69.
7. Organization WH. The World health report: 2005: make every mother and child count. 2005.
8. Kumar S, Singh J. An action plan to assess the current situation of maternal & newborn care at government health facilities in Jharkhand, India. 2007.
9. CSACE I. Ethiopia demographic and health survey 2016. Addis Ababa, Ethiopia, and Rockville, Maryland, USA: CSA and ICF. 2016.
10. Kuhnt J, Vollmer S. Antenatal care services and its implications for vital and health outcomes of children: evidence from 193 surveys in 69 low-income and middle-income countries. BMJ open. 2017;7(11):e017122.
11. Graham W, Woodd S, Byass P, Filippi V, Gon G, Virgo S, et al. Diversity and divergence: the dynamic burden of poor maternal health. The Lancet. 2016;388(10056):2164-75.
12. Organization WH. Neonatal and perinatal mortality: country, regional and global estimates. 2006.
13. d'Ambruoso L, Abbey M, Hussein J. Please understand when I cry out in pain: women's accounts of maternity services during labour and delivery in Ghana. BMC public health. 2005;5(1):140.
14. HSTP F. Health Sector Transformation Plan. Addis Ababa: Federal Ministry of Health (FMOH). 2015.
15. Medhanyie A, Spigt M, Kifle Y, Schaay N, Sanders D, Blanco R, et al. The role of health extension workers in improving utilization of maternal health services in rural areas in Ethiopia: a cross sectional study. BMC health services research. 2012;12(1):1-9.

16. Wang H, Tesfaye R, Ramana GN, Chekagn CT. Ethiopia health extension program: an institutionalized community approach for universal health coverage: World Bank Publications; 2016.
17. Ejigu T, Woldie M, Kifle Y. Quality of antenatal care services at public health facilities of Bahir-Dar special zone, Northwest Ethiopia. BMC health services research. 2013;13(1):443.
18. Huda TM, Chowdhury M, El Arifeen S, Dibley MJ. Individual and community level factors associated with health facility delivery: A cross sectional multilevel analysis in Bangladesh. PloS one. 2019;14(2):e0211113.
19. Yadav A, Kesarwani R. Effect of individual and community factors on maternal health care service use in India: a multilevel approach. Journal of biosocial science. 2016;48(1):1-19.
20. Osorio AM, Tovar LM, Rathmann K. Individual and local level factors and antenatal care use in Colombia: a multilevel analysis. Cadernos de Saúde Pública. 2014;30:1079-92.
21. Kuruvilla S, Bustreo F, Kuo T, Mishra C, Taylor K, Fogstad H, et al. The Global strategy for women's, children's and adolescents' health (2016–2030): a roadmap based on evidence and country experience. Bulletin of the World Health Organization. 2016;94(5):398.
22. Emiru AA, Alene GD, Debelew GT. Women's retention on the continuum of maternal care pathway in west Gojjam zone, Ethiopia: multilevel analysis. BMC pregnancy and childbirth. 2020;20(1):1-14.
23. Medhanyie A, Spigt M, Kifle Y, Schaay N, Sanders D, Blanco R, et al. The role of health extension workers in improving utilization of maternal health services in rural areas in Ethiopia: a cross sectional study. BMC health services research. 2012;12(1):352.
24. Organization WH. Service availability and readiness assessment (SARA): an annual monitoring system for service delivery: reference manual. World Health Organization; 2013.
25. Kianoush F, Masoomehni K. Application REML model and determining cut off of ICC by multi-level model based on Markov Chains simulation in health. Indian Journal of Fundamental and Applied Life Sciences. 2015;5:1432-48.
26. Craney TA, Surles JG. Model-dependent variance inflation factor cutoff values. Quality Engineering. 2002;14(3):391-403.
27. Maheen H, Hoban E, Bennett C. Factors affecting rural women's utilisation of continuum of care services in remote or isolated villages or Pakistan–A mixed-methods study. Women and Birth. 2021;34(3):257-65.

28. Amare NS, Araya BM, Asaye MM. Dropout from maternity continuum of care and associated factors among women in Debre Markos town, Northwest Ethiopia. bioRxiv. 2019:620120.
29. Yeji F, Shibanuma A, Oduro A, Debpuur C, Kikuchi K, Owusu-Agei S, et al. Continuum of care in a maternal, newborn and child health program in Ghana: low completion rate and multiple obstacle factors. PloS one. 2015;10(12):e0142849.
30. Shibanuma A, Yeji F, Okawa S, Mahama E, Kikuchi K, Narh C, et al. The coverage of continuum of care in maternal, newborn and child health: a cross-sectional study of woman-child pairs in Ghana. BMJ global health. 2018;3(4):e000786.
31. Sakuma S, Yasuoka J, Phongluxa K, Jimba M. Determinants of continuum of care for maternal, newborn, and child health services in rural Khammouane, Lao PDR. PloS one. 2019;14(4):e0215635.
32. Asratie MH, Muche AA, Geremew AB. Completion of maternity continuum of care among women in the post-partum period: Magnitude and associated factors in the northwest, Ethiopia. PloS one. 2020;15(8):e0237980.
33. Atnafu A, Kebede A, Misganaw B, Teshome DF, Biks GA, Demissie GD, et al. Determinants of the Continuum of Maternal Healthcare Services in Northwest Ethiopia: Findings from the Primary Health Care Project. Journal of Pregnancy. 2020;2020.
34. Haile D, Kondale M, Andarge E, Tunje A, Fikadu T, Boti N. Level of completion along continuum of care for maternal and newborn health services and factors associated with it among women in Arba Minch Zuria woreda, Gamo zone, Southern Ethiopia: A community based cross-sectional study. Plos one. 2020;15(6):e0221670.
35. Chaka EE, Parsaeian M, Majdzadeh R. Factors associated with the completion of the continuum of care for maternal, newborn, and child health services in Ethiopia. Multilevel model analysis. International Journal of Preventive Medicine. 2019;10.
36. Tizazu MA, Sharew NT, Mamo T, Zeru AB, Asefa EY, Amare NS. Completing the Continuum of Maternity Care and Associated Factors in Debre Berhan Town, Amhara, Ethiopia, 2020. Journal of Multidisciplinary Healthcare. 2021;14:21.
37. Mohan D, LeFevre AE, George A, Mpembeni R, Bazant E, Rusibamayila N, et al. Analysis of dropout across the continuum of maternal health care in Tanzania: findings from a cross-sectional household survey. Health policy and planning. 2017;32(6):791-9.
38. Singh K, Story WT, Moran AC. Assessing the continuum of care pathway for maternal health in South Asia and sub-Saharan Africa. Maternal and child health journal. 2016;20(2):281-9.

39. Iqbal S, Maqsood S, Zakar R, Zakar MZ, Fischer F. Continuum of care in maternal, newborn and child health in Pakistan: analysis of trends and determinants from 2006 to 2012. BMC health services research. 2017;17(1):189.
40. Chalise B, Chalise M, Bista B, Pandey AR, Thapa S. Correlates of continuum of maternal health services among Nepalese women: Evidence from Nepal Multiple Indicator Cluster Survey. PLoS One. 2019;14(4):e0215613.
41. Kikuchi K, Yasuoka J, Nanishi K, Ahmed A, Nohara Y, Nishikitani M, et al. Postnatal care could be the key to improving the continuum of care in maternal and child health in Ratanakiri, Cambodia. PloS one. 2018;13(6):e0198829.
42. Wang W, Hong R. Levels and determinants of continuum of care for maternal and newborn health in Cambodia-evidence from a population-based survey. BMC pregnancy and childbirth. 2015;15(1):1-9.
43. Tamang TM, editor Factors associated with completion of continuum of Care for Maternal Health in Nepal. IUSSP XXVIII International Population Conference, Cape Town, South Africa; 2017.
44. Hamed A, Mohamed E, Sabry M. Egyptian status of continuum of care for maternal, newborn, and child health: Sohag Governorate as an example. International Journal of Medical Science and Public Health. 2018;7(6):1.
45. Gebremeskel F, Dibaba Y, Admassu B. Timing of first antenatal care attendance and associated factors among pregnant women in Arba Minch Town and Arba Minch District, Gamo Gofa Zone, South Ethiopia. Journal of environmental and public health. 2015;2015.
46. Tsegay Y, Gebrehiwot T, Goicolea I, Edin K, Lemma H, San Sebastian M. Determinants of antenatal and delivery care utilization in Tigray region, Ethiopia: a cross-sectional study. International journal for equity in health. 2013;12(1):1-10.
47. Yaya S, Bishwajit G, Ekholuenetale M, Shah V, Kadio B, Udenigwe O. Timing and adequate attendance of antenatal care visits among women in Ethiopia. PLoS One. 2017;12(9):e0184934.
48. Agha S, Tappis H. The timing of antenatal care initiation and the content of care in Sindh, Pakistan. BMC pregnancy and childbirth. 2016;16(1):1-9.
49. Khatib N, Zahiruddin Q, Gaidhane A, Waghmare L, Srivatsava T, Goyal R, et al. Predictors for antenatal services and pregnancy outcome in a rural area: a prospective study in Wardha district, India. Indian journal of medical sciences. 2009;63(10):436.
50. Gall A, Shenkute Z, Kiefer D, Jackson J. Ethiopian traditional and herbal medications and their interactions with conventional drugs. Ethno Med. 2009;1:1-4.

51. Paul BK, Rumsey DJ. Utilization of health facilities and trained birth attendants for childbirth in rural Bangladesh: an empirical study. Social science & medicine. 2002;54(12):1755-65.
52. Fekadu GA, Ambaw F, Kidanie SA. Facility delivery and postnatal care services use among mothers who attended four or more antenatal care visits in Ethiopia: further analysis of the 2016 demographic and health survey. BMC pregnancy and childbirth. 2019;19(1):1-9.
53. Bulambo HK. Utilization of Maternal Health Care Services in Rural Mali: The Role of Traditional Values: Emory University; 2012.
54. Zepre K, Kaba M. Birth preparedness and complication readiness among rural women of reproductive age in Abeshige district, Guraghe zone, SNNPR, Ethiopia. International journal of women's health. 2017;9:11.
55. Damtew ZA, Karim AM, Chekagn CT, Fesseha Zemichael N, Yihun B, Willey BA, et al. Correlates of the Women’s Development Army strategy implementation strength with household reproductive, maternal, newborn and child healthcare practices: a cross-sectional study in four regions of Ethiopia. BMC pregnancy and childbirth. 2018;18(1):5-17.
56. Ftwi M, Gebretsadik GG-e, Berhe H, Haftu M, Gebremariam G, Tesfau YB. Coverage of completion of four ANC visits based on recommended time schedule in Northern Ethiopia: A community-based cross-sectional study design. PloS one. 2020;15(8):e0236965.
57. Tesfau YB, Kahsay AB, Gebrehiwot TG, Medhanyie AA, Godefay H. Postnatal home visits by health extension workers in rural areas of Ethiopia: a cross-sectional study design. BMC Pregnancy and Childbirth. 2020;20:1-9.
58. Arthur E. Wealth and antenatal care use: implications for maternal health care utilisation in Ghana. Health economics review. 2012;2(1):1-8.
59. Manzi A, Munyaneza F, Mujawase F, Banamwana L, Sayinzoga F, Thomson DR, et al. Assessing predictors of delayed antenatal care visits in Rwanda: a secondary analysis of Rwanda demographic and health survey 2010. BMC pregnancy and childbirth. 2014;14(1):1-8.
60. Aliyu AA, Dahiru T. Predictors of delayed Antenatal Care (ANC) visits in Nigeria: secondary analysis of 2013 Nigeria Demographic and Health Survey (NDHS). The Pan African medical journal. 2017;26.
61. Kibusi SM, Sunguya BF, Kimunai E, Hines CS. Health insurance is important in improving maternal health service utilization in Tanzania—analysis of the 2011/2012 Tanzania HIV/AIDS and malaria indicator survey. BMC health services research. 2018;18(1):1-10.

62. Agha S, Carton TW. Determinants of institutional delivery in rural Jhang, Pakistan. International journal for equity in health. 2011;10(1):31.
63. Health FMo. Rural Community Health Information System implementation Manual. Lancet. 2019;Vol. 372.
64. Gilmore B, McAuliffe E. Effectiveness of community health workers delivering preventive interventions for maternal and child health in low-and middle-income countries: a systematic review. BMC public health. 2013;13(1):1-14.
65. Dadi LS, Berhane M, Ahmed Y, Gudina EK, Berhanu T, Kim KH, et al. Maternal and newborn health services utilization in Jimma Zone, Southwest Ethiopia: a community based cross-sectional study. BMC pregnancy and childbirth. 2019;19(1):1-13.
66. Gabrysch S, Cousens S, Cox J, Campbell OM. The influence of distance and level of care on delivery place in rural Zambia: a study of linked national data in a geographic information system. PLoS medicine. 2011;8(1):e1000394.
67. Kenny A, Basu G, Ballard M, Griffiths T, Kentoffio K, Niyonzima JB, et al. Remoteness and maternal and child health service utilization in rural Liberia: A population–based survey. Journal of global health. 2015;5(2).
68. Choulagai B, Onta S, Subedi N, Mehata S, Bhandari GP, Poudyal A, et al. Barriers to using skilled birth attendants' services in mid-and far-western Nepal: a cross-sectional study. BMC international health and human rights. 2013;13(1):1-9.
69. Abera M, Belachew T. Predictors of safe delivery service utilization in Arsi Zone, South-East Ethiopia. Ethiopian journal of health sciences. 2011;21(3).
70. Sharkey JT, Puttaramu R, Word RA, Olcese J. Melatonin synergizes with oxytocin to enhance contractility of human myometrial smooth muscle cells. The Journal of Clinical Endocrinology & Metabolism. 2009;94(2):421-7.
71. Glei DA, Goldman N, Rodríguez G. Utilization of care during pregnancy in rural Guatemala: does obstetrical need matter? Social science & medicine. 2003;57(12):2447-63.
72. Berhan Y, Berhan A. Commentary: reasons for persistently high maternal and perinatal mortalities in Ethiopia: part III–perspective of the "three delays" model. Ethiopian journal of health sciences. 2014;24:137-48.

Figure Click here to access/download;Figure;Fig 1.tif

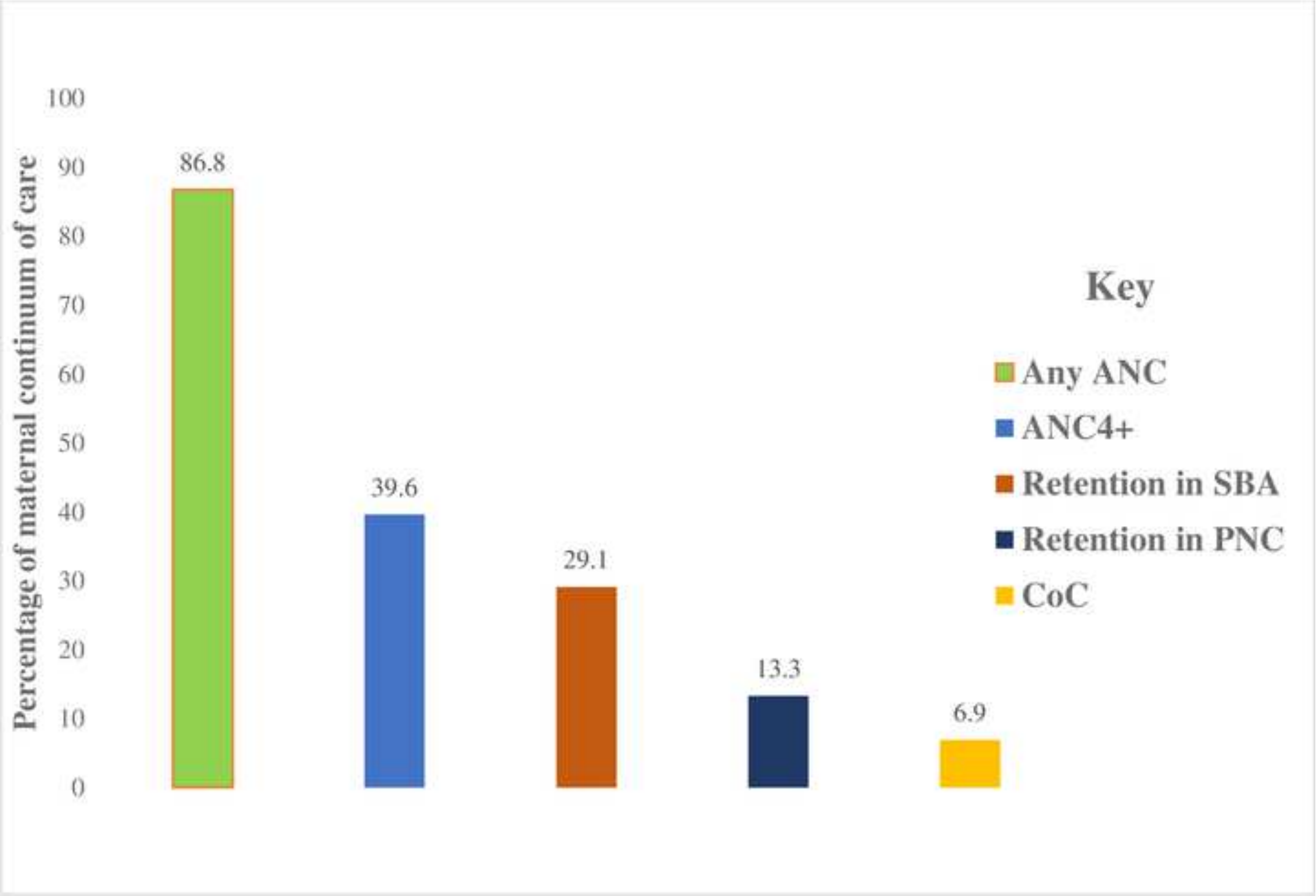

Supplement: Attachment — Submitted filename: PONE-D-21-38409_reviewer_comments.pdf [file pone.0274729.s002.pdf]
