# Supplementary material for: Why maternal continuum of care remains low in Northwest Ethiopia? A multilevel logistic regression analysis
Source: PLoS One. 2022 Sep 19;17(9):e0274729. doi: 10.1371/journal.pone.0274729 (PMC9484641; doi:10.1371/journal.pone.0274729)
Supplement: Attachment — Submitted filename: PONE-D-21-38409_reviewer comments.docx [file pone.0274729.s003.docx]

**Overall impression**

The paper is clearly and logically written, well organized, and easy to follow. The introduction provides the reader useful background and context that sets up the rest of the paper well. The authors present robust and novel findings that address the factors that affect women’s completion of the maternal continuum of care in the Wogera and Gondar districts through a community-based cross-sectional study. The methods were clearly defined, and the results and discussions well outlined and supported with existing evidence and past studies in Ethiopia. The paper also did well to adjust for the design effect by assessing various levels of intraclass correlation coefficients using four different models.

The paper requires major and minor revisions including the revision of wording choice and consistency, the justification of the interview tool and sampling technique, addition of citations, and the expansion on the discussion of study limitations. Moreover, I would advise the author to complete a grammar revision of the paper to improve the flow and readability of the text and revise the structure of some of the sections within the paper.

Please refer to the minor revisions for detailed recommendations.

**Minor revisions:**

1. The paper needs more discussion of study limitations which may include expanding on:
   - Selection bias: error due to random chance, bias from selecting the 11 health centers (clusters) and kebeles for the study; limited external validity: study only conducted in 2 districts in Ethiopia
   - Confounding bias: any other unknown or unmeasured confounding factors that were not controlled for

**Minor revisions:**

| **Paper Section** | **Suggestion** | **Specific example** |
| --- | --- | --- |
| Overall | Keep the word choice and labelling consistent | Utilize relevant and defined acronyms consistently throughout paper:   - Page 3, Line 17: antenatal care was defined as ANC. Suggest using “ANC” consistently throughout rest of the paper - Suggest assigning acronym “TBA” to ‘Traditional birth attendants” earlier in the paper and standardizing use of “TBA” throughout the rest of paper - Suggest assigning acronym “SBA” to “Skilled birth attendants” early in the paper and standardizing use of “SBA” throughout rest of paper - Suggest assigning acronym “WDA” to “Women’s Development Army” earlier in the paper, as Table 3 references “WDA” without the acronym being defined earlier and standardizing use of WDA in the rest of paper - Suggest assigning acronym “HEW” to “Health Extension Worker” earlier in the paper (before page 20) since Table 3 references ‘HEW” without the acronym being defined earlier and standardizing use of HEW throughout the rest of the paper - Suggest assigning acronym “PNC” to postnatal care and standardizing use of PNC throughout the rest of the paper - Intraclass correlation was assigned acronym “ICC” on Page 9, Line 12. No need to define the acronym again on Page 9, Line 21. Suggest using “ICC” consistently throughout rest of the paper - Suggest revising “antenatal care four and above” to “at least 4 ANC visits”   - Page 5, Lines 23-24   - Page 8, Lines 12-13   - Page 13, Line 5   - Page 14, Line 1   - Page 18, Line 10 |
| Abstract | Word choice | - Page 2, Line 3: suggest replacing “intractable” to “significant” |
| Introduction | Wording clarification and consistency | Suggest revising the following sentences:   - Page 3, Line 4-6: clearly explain what the numbers represent (e.g., number of deaths, life-time risk). Also, please clarify which year these statistics were estimated in. - Page 3, Line 10: suggest adding year of estimation. - Page 5, Line 3-4: please clarify what you mean by “women with less than one year” – what is the less than one year referring to?   Wording choice and consistency:   - Page 4, Line 1: suggest changing “skilled healthcare attendant” to “skilled birth attendant” (SBA) to align with wording in rest of paper - Page 4, Line 11: suggest changing “besides” to “moreover” - Page 4, Line 16: suggest revising “studies” to “past/recent studies” |
| Methods | Sample size clarification | Consider including a sample size calculation table in Appendix:   - Suggest presenting sample size calculation matrix table under different scenarios (and assumptions) and justification of the chosen sample size |
|  | Clarify age range | Page 5, line 14: Please clarify what the age range for reproductive age group is (e.g., 15-54) and explain why this range was chosen. |
|  | Clarify outcome variable | Page 9, Line 3:   - Suggest clarifying that the outcome variable is the “maternal continuum of care” (binary) |
| Results | Revise tables | Tables 1-2:   - Suggest removing column “Total (%)”: it is not contributing more useful information   Tables 1-3:   - Make sure that spacing is consistent in table 1 (e.g., N (%)) |
|  | Wording choice | Table 1:   - Page 11, suggest revising “intendedness of pregnancy” to “pregnancy intention”   Wording choice and consistency:   - Standardize capitalization of “Model I-IV” in Results section,   - Page 14, Line 22: Capitalize “model II”   - Page 15, Line 1: Capitalize “model III”   - Page 15, Line 4: Capitalize “model IV” - Clarify “counterparts” when summarizing Odds Ratios and comparisons   - Suggest revising “counterparts” to “lower-education counterparts” (Page 15, Line 11) |
|  | Missing results | - Page 14, Line 17: fill in the blank: what = 0.43? |
| Discussion | Add citations | - Page 17, Line 3-4: add citations for statistics “one of every fourteen” and “one out of eight” - Page 19, Lines 16, 19-20: add citations for the community-based health insurance scheme/reform in Ethiopia |
|  | Wording choice and consistency and clarification | Wording choice and consistency:   - Suggest revising Page 18, Lines 12-13 to “puts women at higher risk of unwanted death” - Suggest revising Page 19, Lines 18-19 to “aligns”   Wording clarification:   - Please clarify what “elsewhere” means (Page 19, Line 19) |
|  | Clarification needed | Please justify why Pakistan and other countries (e.g., Cambodia, Nepal, Lao PDR, Egypt) were included as comparisons of interest to the results of the study. Would suggest removing these comparisons and only keeping references in Ethiopia. |
|  | Strengths & Limitations | Strengths: No strengths were outlined in the Discussion section  Limitations: to consider further limitations in the study   - Consider selection bias: error due to random chance, bias from selecting the 11 health centers (clusters) and kebeles for the study; limited external validity: study only conducted in 2 districts in Ethiopia. Explain the relevance of the study beyond the context of Ethiopia. - Consider confounding bias: any other unknown or unmeasured confounding factors that were not controlled for - Study instrument: clarify whether tool is standardized in Ethiopia / beyond; explain who the “expert reviewers” were |
| Grammar | Grammar | Please revise comments throughout manuscript (attached PDF). |
